# Impact of Immediacy of Feedback on Continuous Intentions to Use Online Learning From the Student Perspective

**DOI:** 10.3389/fpsyg.2022.865680

**Published:** 2022-06-30

**Authors:** Rong Yu, Xuerui Cai

**Affiliations:** ^1^College of Art and Design, Shangqiu Normal University, Shangqiu, China; ^2^School of Media and Communication, Wuhan Textile University, Wuhan, China

**Keywords:** immediacy of feedback, technology acceptance model, information system continuance, online learning environment, COVID-19

## Abstract

The immediacy of feedback in media is emerging to enhance the interactivity of online experience for users. There is a gap in the study to explore the impacts of the immediacy of feedback on continuous intentions to use online learning from the student perspective. This study aims to fill the gap to investigate the impacts of the immediacy of feedback on students’ continuous intentions to use online learning. This study utilizes the technology acceptance model (TAM) and expectation theory model (ETM) to conceptualize the effect of the immediacy of feedback on student continuous intentions to use online learning in terms of the mediation effect of Perceived Ease of Use (PEOU), Perceived Usefulness (PU), satisfaction, and attitude of students for continuous intentions to use online learning. An online survey of higher education students with experience in online learning is conducted to test the proposed hypothesis. The collected data are analyzed by using structural equation modeling (SEM) to establish the proposed hypothesis. The findings reveal that the immediacy of feedback from the media has a strong association with PEOU, PU, students’ attitudes, students’ satisfaction, and ultimately toward the continuous intentions to use online line learning in future. The study set key theoretical and practical insights to pave the way for future research.

## Introduction

Information and communication technologies (ICTs) have brought a revolution in every field of life, including education in the form of online learning. Online learning is the modern distance mode of learning. Online learning is a distance mode of learning that traces back to 1800 to Isaac Pitman, who uses letters to teach students. Online learning has gained popularity and recognition for distributing educational resources across the world (Business School and Ali, 2017; Kurdi et al., 2020). Online learning has gained fame and appreciation in dispensing educational resources across the world (Abdul Rahman et al., 2020). It is proclaimed that online learning would replace the traditional model of education in future due to the advantages associated with online learning. Online learning is the use of modern communication technologies to conduct distance modes of synchronous or asynchronous learning systems (Huang, 2021).

With the advancement in media richness, students can experience interaction and receive immediate feedback. Media richness capabilities provide more interaction and immediacy of feedback to and from students. Online learning is a modern way of E-learning (Huang, 2021). Govindasamy (2001) believes that the emergence of satellite communication, the Internet, system user interactivity, and modern storage media like compact drives (CD) has created a new type of learning environment known as digital learning. To break through times and overcome the difference in time zones, the asynchronous mode of online learning is more popular. Much research was conducted to describe the usefulness of the online system, but the online learning industry does not feel such acceptance and expansions in online learning.

The advancements in technology have added richness to the media that offers excitement to online learning. The media richness has empowered the distance education mode to online learning that online courses are a core part of many institutes across the world (Govindasamy, 2001). Depending on the demands and requirements, online learning can be synchronous, synchronous, collaborative, or corporate. In collaborative learning, the peers share and learn with each other. Education institutes across the world also take advantage of online learning to reach a new market. Students, on the contrary, are also interested in online learning due to the inherent advantage of cost, and access to knowledge in their space and time. Due to these two-sided advantages, online learning is increasing day by day into different shapes. Moreover, with advancements in ICT and an increase in media richness, the effectiveness of online learning is profound (Huang, 2021; Zhou et al., 2021).

Besides, the tremendous growth of online learning has reached 17% per annum. With this tremendous growth, the failure of online learning is also observed in different prospects like acceptance by students and continuous usage intentions (Business School and Ali, 2017). The success of online learning depends upon many factors like acceptance from students, the attitude of students toward online learning the usefulness of online learning. Different studies were conducted to assess the continuous intentions of online learning with different factors. Zheng (2022) identified the cognitive and affective factors that are important to the success of online learning with the help of underlying constructs. Mehta and Shah (2022) investigated the student perception of online learning as compared to face-to-face learning. Setiyawan and Santoso (2022) explored the acceptance of online learning by students of higher education in Indonesia. Azi et al. (2022) explored the effectiveness of online learning for the improvement of core competencies in medical training related to fracture-related infestation.

Studies also investigated the impacts of different types of interactions on the success of online learning (York and Richardson, 2017). The immediacy of feedback in media is an important attribute of media richness that can cause the success of online learning that is not explored by any researcher. The immediacy of feedback is an important attribute for continuous intentions to use online learning that needs to be investigated for the success of online learning. There is a need to assess the impacts of the immediacy of feedback from media on students’ continuous intentions to use online learning directly as well as by mediating effects like the ease of use, usefulness, student attitude toward online learning, and student satisfaction with online learning. There is no existing study that explores the impacts of the immediacy of feedback on student’s continuous intentions to use online learning with the integration of multiple theories like the technology acceptance model (TAM) and information system continuance (IS continuance). To fill the gap, the study explored the impact of the immediacy of feedback from media on the continuance intentions to use online learning with the integration of TAM and IS continuance theories. The study explores the desired relationship through Perceived Ease of Use (PEU), satisfaction, attitude, Perceived Usefulness (PU), and students’ problem-solving skills.

## Theoretical Background

### Media Richness Theory

**FIGURE 1:**
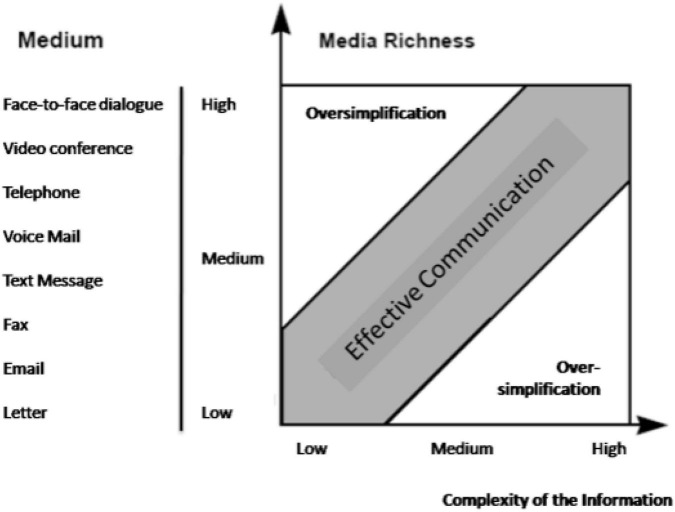
Media richness of different media (Daft and Lengel, 1986).

The media richness theory shown in Figure 1 proposed by Daft and Lengel (1986) elaborates on the media’s ability to reproduce the information (Dennis and Kinney, 1998). Each communication media have certain visual and social cues that describe the communication media and are used to rank the evaluation of the media. For example, telephone, E-mail, and video conferences have different visual social cues. Telephone calls cannot show the gesture and body language of the participants and are perceived as less rich as compared to video conferences. MRT theorizes that rich media are more effective in communication of equivocal issues as compared to the lesser rich media. Media richness is defined by “the ability of information to change understanding within a time interval.” The theory is used to evaluate the media based on their abilities to communicate messages and change understanding. Media that can convey ambiguous issues in lesser time as compared to the lesser rich media require more time to covey same understanding. The primary concern for selecting the media is the reduction of possible misinterpretation of the conveyed message. Media richness theory differentiates the media into rich and lean based on the authenticity of the sending of the message over the medium (Timmerman and Kruepke, 2006).

Media richness is defined based on the number of channels, immediate feedback, and level of personal communication achieved with the media. The ability of the media to facilitate understanding, effectively dealing interpretations, and resolve ambiguity makes a media lean or rich. The number of channels is described by tone, gesture, and tone are the major elements. Face-to-face communication is richer in the context of the number of channels. In context media, the video conference is much richer as compared to E-mail, text messages, and audio calls (Timmerman and Kruepke, 2006).

The immediacy of feedback from media refers to the ability of the media to enable the receiver to provide feedback on the received communication. The sender and receiver are the two important pillars of communication (Barber, 2018). For effective communications, the receiver intends to understand the message intended. The receiver’s ability to provide feedback improves the understanding of the message. The feedback is classified as concurrent and sequential. Concurrent feedback is the feedback the sender provides with the message, and concurrent feedback is in the form of non-verbal clues like nodding and expressions. Feedback plays important role in effective communication because it helps to adjust the sender to reorganize the message according to the receivers and moves as per the understanding of the receiver. Immediate feedback is also helpful to reorganize the patterns to reduce the communication time. One approach in this regard is the use of the installments in message delivery according to the user’s understanding. It is observed that immediate feedback improves the overall effectiveness of communication by reducing the time to communicate. Dennis and Kinney (1998) proved that media richness has an impact on performance in the team when assigned equivocal tasks. The performance of the team member in making decisions improved with richer media.

#### Technology Acceptance Model

The technology acceptance model (TAM) (Davis, 1989) aims to model the user behavior toward a system in terms of ease of use of the system and usefulness of the system. PEU and PU are determinants of attitude that ultimately determine the intentions to use technology. The PEU is also a direct determinant of the intentions to use technology. Intention to use technology determines the actual usage of the technology. Perceived Ease of Use (PEU) is the major determinant of attitude toward technology, and intentions to use online learning. The use of multimedia technologies increases the effectiveness and usefulness of E-learning systems (Chai et al., 2013).

Al-Gahtani (2016) explored the continuous intentions to use online learning using TAM3. Fathema (2015) explored the factors that affect the faculty’s attitude and behavior toward the use of the learning management system (LMS). According to the study Perceived Usefulness (PU), perceived self-efficacy and facilitation are the important factors that affect the faculty behavior toward the LMS. Ashrafi et al. (2020) proposed an integrated model to observe the perceived enjoyment and social influence on continuous intention to use LMS. Weng et al. (2015) proposed a framework to explain learning satisfaction with continuous intentions to use an online system in organization training. The study finds that supervisors, peers, and family support have positive support for employee learning satisfactionClick or tap here to enter text.

Kurdi et al. (2020) proposed a theoretical framework founded on the TAM model. According to the study, the Perceived Ease of Use (PEU) Perceived Usefulness (PU), self-efficacy, perceived enjoyment, and social influence are the important factors that affect the student’s intentions toward E-learning (Kurdi et al., 2020). Fathema (2015) explored the factors that affect the faculty’s attitude and behavior toward the use of the learning management system (LMS). According to the study, Perceived Usefulness (PU), perceived self-efficacy, and facilitation are the important factors that affect the faculty behavior toward the LMS (Fathema, 2015). Kurdi et al. (2020) proposed a theoretical framework founded on the TAM to identify the important factors that affect the student’s intentions toward E-learning.

#### Expectation–Confirmation Theory

Oliver (1976, 2018) proposed the user satisfaction after a product use in terms of expectation–confirmation theory (ECT). According to the ECT theory, four constructs named “expectations, perceived performance, disconfirmation of beliefs, and satisfaction” affect the user’s intentions to continuously use a product (Oliver, 1976). Bhattacherjee (2001) extended ECT theory to a user decision of continuous use of information systems (IS). The IS continuance model describes the user experience after the use of an information system (IS). According to the IS continuance theory, continuous usage intention is an important factor that determines the success of the technology. The strength of continuous intention to use technology affects the user’s decision to continuously use technology or not (Alraimi et al., 2015). Lee (2010) proposed a model to predict the user behavior toward continuously using E-learning. Huang (2021) explores the factors that can affect the student’s continuous usage of online learning from PEU, PU, and social capital.

Although online learning is very common and popular, it also suffers from disconsolation. The factors that can affect the continuous intentions to use online learning are important (Lee, 2010). Paraskeva et al. (2010) recommend multiplayer online games to enhance collaboration among students. The study is based on activity theory. Oghuma et al. (2016) proposed the continuous intention to use mole messaging using the expectation–confirmation model (ECM). Al-Adwan et al. (2021) proposed a sustainable E-learning model using the structural equation model. Panigrahi et al. (2021) explore the effectiveness of E-learning by students mediating the role of student engagements. Persada et al., 2021 proposed student continuous intentions to use online tutoring by expectation–confirmation model (ECM). Cheng (2021) examined the E-learning in a medical professional for continuous intentions by assessment of the perceived impact of learning on perceived tasks in medical institutes. The study analyzes the impact of cognitive absorption (CA) through learning technology fit (LTF) and task technology fit (TTF).

Paraskeva et al. (2010) presented a multiplayer educational game to promote collaborations among students. Mellikeche et al. (2020) evaluated the Unified Model of Information System Continuance (UMISC) to evaluate the clinical information system. According to the study, the UMISC model is a robust model to observe the continuance intentions to use and satisfaction in post-adaptation of a clinical information system (Mellikeche et al., 2020). Gupta et al. (2020) recommended a model for determining the impacts of post-adaptation confirmation and expectancies on satisfaction and continuous intentions. According to the study, self-efficacy, satisfaction, and perceived usefulness are strong antecedents of continuous intentions to adopt a technology (Gupta et al., 2020). The related work is also summarized in Table 1.

**TABLE 1:** Related work.

Research context	Foundation theories	Constructs	Ref.
E-learning	TAM, TPB, ECT, Flow theory	Attitude, Ease of Use, Behavioral Control, Concentration, Enjoyment, Continuous Intentions	Lee, 2010
MOOCs	ECT, Flow Theory	Perceived Usefulness, Confirmation, Satisfaction, Continuous Intention	Lu et al., 2019
MOOCs	ECT	Attitude, Curiosity, Continuance Intentions, Satisfaction Usefulness, Confirmation	Dai et al., 2020
MOOCs	ECT	Continuance Intentions, Performance proficiency, Knowledge outcome, Confirmation, Satisfaction, Social Influence	Junjie, 2017
MOOCs	ECT	Openness, Reputation, Enjoyment, Continuance intention, Satisfaction, Usefulness, Confirmation	Alraimi et al., 2015
Online learning	Task Technology Fit (TTF), ECT	Confirmation, Usefulness, Satisfaction, Continuance Intentions, Task Technology Fit (TTF)	Wang et al., 2021

## Research Model and Hypotheses

The study wants to explore the impact of relative immediacy of feedback of media richness attribute on the continuous intentions to use online learning. The motivation of the study is to explore the impacts of media richness on the adaptation and acceptance of the online learning system by students to determine the future of the online education industry and directions for educational institutes.

### Research Model and Hypotheses

**FIGURE 2:**
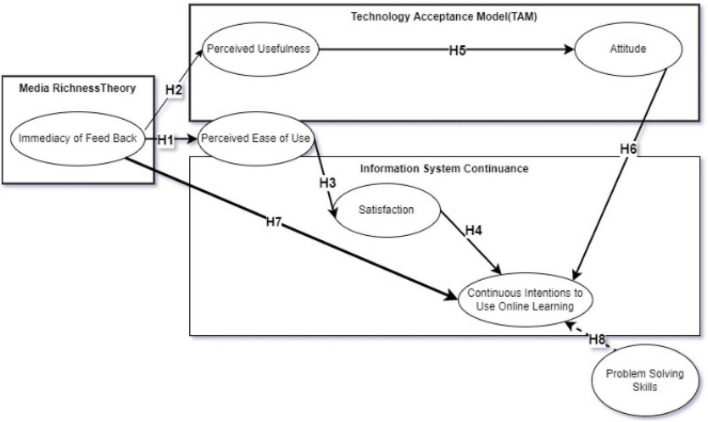
Conceptual model.

Media richness theory and expectation–confirmation theory are used to find the relationship between the immediacy of feedback from media to the continuous intentions of using an online learning system. The direct relationship between the immediacy of feedback attribute of media and continuous intentions of using the E-learning system is determined by an intermediate relationship between perceived usefulness of the E-learning system and user satisfaction with the online learning system. The research model is shown in Figure 2 with Hypotheses H1 to H8. Hypotheses H1, H3, H4, and H5 are derived from IS continuance theory and technology acceptance model (TAM). The hypotheses H2, H6, and H7 are derived from TAM theory. These constructs are well-known antecedents of continuous intentions of information systems. The study wants to explore the impacts of the immediacy of feedback on the continuous intentions to use online learning systems with the help of these antecedents’ constructs. The impacts of student online skills on the continuance of online learning are also explored as moderators.

### Research Hypothesis

The proposed study intends to explore the impacts of media richness with the immediacy of feedback on the continuous intentions of the E-learning system. TAM was extensively applied in exploring the factors that affect the success of online learning in terms of users’ continuous intentions to use online learning (Kurdi et al., 2020). According to the TAM, the PEOU and PU are two important elements that affect the user perceptions of the acceptance of new technologies (Davis, 1989). PU is the user’s perception that using technology would help them to achieve more advantages (Lu et al., 2019). On the contrary, PEOU is the perception that using the new technology would be simple and uncomplicated. Rather than spending time learning about the system, the user would feel to use it without extra effort and time (Davis, 1989). The user-friendly system creates more PEOU (Austermann and Mertins, 2014). PU and PEOU are very well associated with the success of the online learning system (Al-Rahmi et al., 2019). Based on the media richness theory, IS continuance theory, and TAM theory, hypotheses 1 and 2 are made to analyze the impact of the immediacy of feedback on students’ Perceived Ease of Use (PEU) of online learning systems.

**Hypothesis 1 (H1):** Immediacy of feedback in online learning is positively correlated with the student Perceived Ease of use (PEU) of online learning

**Hypothesis 2 (H2):** Immediacy of feedback in online learning is positively correlated with the student Perceived usefulness (PU) of the online learning

Satisfaction is one of the important parameters that determine the success in terms of its future adaptation (Liao et al., 2022). Satisfaction is considered an important element of online learning that students experience and determine the value of the experience (Mehta and Shah, 2022). Satisfaction is the feeling of the experience that altered the inclination of the user toward the system usage. Perceived Ease of Use (PEOU) experience with the use of the system affects the user satisfaction with the system (Setiyawan and Santoso, 2022). The student’s perception that online learning is easy to use is positively associated with students’ satisfaction with online learning (Sugiarta et al., 2022). A satisfied user is more inclined toward continuous use of a particular system. PEOU experience of students with online learning the students tend to be inclined toward the continuous intentions to use online learning in future (Alraimi et al., 2015). Based on IS continuance theory, hypotheses 3 and 4 are made to analyze the impact of student Perceived Ease of Use (PEU) on students’ satisfaction with the online learning system.

**Hypothesis 3 (H3):** Student Perceived Ease of Use (PEU) of online learning system is positively correlated with the student’s satisfaction with online learning

**Hypothesis 4 (H4):** Student satisfaction is positively correlated with students’ continuous intentions to use an online learning system

Attitude is shaped by students’ experience and assessment of the system in terms of PU of the online learning system (Austermann and Mertins, 2014). PEOU and PU channel the attitude of the user toward online learning (Bhattacherjee, 2001). PEOU and PU are the key elements that determine the attitude of the students toward the online system (Sugiarta et al., 2022). The student’s attitude toward the online learning system determines the student’s continuance intentions to use online learning (Dai et al., 2020). The possibilities of the use of a system are observed in terms of future intentions to adopt a system. The user has intentions to acquire a product or service only when they sense the PEOU and PU from the usage of that product or service (Dai et al., 2020). Users shift their attitude and satisfaction based on the PU and PEOU. Based on IS continuance theory, hypotheses 5 and 6 are made to analyze the impact of student attitude toward the online learning system on ultimately to the students’ continuous intentions to use online learning. Based on TAM theory, hypothesis 6 is made to analyze the impact of student attitude toward the online learning system on students’ continuous intentions to use online learning.

**Hypothesis 5 (H5):** Perceived Usefulness (PU) of online learning system is positively correlated with the student’s attitude toward online learning

**Hypothesis 6 (H6):** Students’ attitudes toward online systems is positively correlated with student’s continuous intentions to use online learning

In the case of online learning, the student’s satisfaction and attitude toward the online learning system would determine the students’ intentions toward the online system (Al-Rahmi et al., 2019). Many studies were conducted to explore the interaction models that are important for the success of online learning (Alebeisat et al., 2022). To determine the direct relationship between the immediacy of feedback from media and students’ continuance intentions to use online learning, hypothesis 7 is made.

**Hypothesis 7 (H7):** Immediacy of feedback from media is positively correlated with students’ continuous intentions to use online learning

Students’ interpersonal skills have subtle impacts on the adaptation of new technologies in the form of online learning (Çevik et al., 2015). Different studies were conducted to explore the impacts of online learning on student problem-solving skills (Giyanto et al., 2020). Different factors of success of online learning were extensively identified by many researchers (Habes et al., 2022). Problem-solving skills of students are an important attribute of online learning to continuously use online learning in future (Giyanto et al., 2020). To determine the impact of students’ problem-solving skills on continuous intentions to use online learning with the immediacy of feedback, hypothesis 8 is made.

**Hypothesis 8 (H8):** Student problem-solving skills be positively correlated to enhance the immediacy of feedback of media relationship with students’ continuous intentions to use online learning

### Definitions of Constructs

The operational definition of the construct is defined in Table 2, with their source.

**TABLE 2:** Operational definition of constructs.

Constructs	Operational definition	Source
The immediacy of feedback from media	The capacity of the media to allow participants to provide feedback immediately	Kahai and Cooper, 2003; Fajfar et al., 2012
Perceived usefulness	The level to which a technology is helpful in the completion of a task, as compared to existing solutions	Davis, 1989; Bhattacherjee, 2001
Satisfaction	The degree to which users are satisfied with the technology	Bhattacherjee, 2001
Continuous usage intentions	The degree to which user’s behavioral tendency to adopt online learning in the future	Davis, 1989; Bhattacherjee, 2001; Cheng, 2021
Attitude	My personal feeling about a technology	Bhattacherjee, 2001
Perceived Ease of Use	The level to which a technology is easy to use as compared to existing	Davis, 1989

## Methodology

To find the impacts of the immediacy of feedback on students’ continuous intentions to use online learning, a survey was conducted among students with experience with online learning. The objective of this investigation is to explore the impacts of the immediacy of the feedback feature of media on the success of online learning from the students’ perspective.

### Study Instrument

A survey instrument is used to establish the hypotheses of the study. Different items for each construct of the study are used and given in Table 3. The identified constructs are supported by theoretical background.

**TABLE 3:** Constructs with items.

Constructs	Number of items	Source
Perceived Ease of Use (PEU)	4	Davis, 1989
Satisfaction (SAT)	3	Oliver, 1976, 2018; Bhattacherjee, 2001
Attitude (AT)	3	Oliver, 1976, 2018; Bhattacherjee, 2001
The immediacy of Feedback (IF)	3	Bhattacherjee, 2001; Kahai and Cooper, 2003; Fajfar et al., 2012
Continuous Intentions to use online Learning (CIOL)	3	Bhattacherjee, 2001; Chen et al., 2021
Perceived Usefulness (PU)	4	Davis, 1989; Cheng, 2021

### Participants

The sample population of the university students enrolled in the graduate or postgraduate level who have experience of at least one semester in the online learning system are selected. The students of developing countries enrolled in a higher education degree program with experience in online learning are the target population of the study. The participants of the study are selected who have experience with Zoom, Microsoft Teams, and WebEx. The participants have been selected who have experience in online learning for at least one semester.

#### Demographics Data of Participants

This section describes the demographic data about responding students. The survey is conducted using the Internet with the help of the google form. The survey is distributed among students with 1118 responses. The survey is conducted by students studying in Chinese universities. The survey is taken immediately after COVID-19, and students have sufficient experience with online learning. The survey is conducted from September to October 2021. It is observed out of the total respondents, 53.5% were female and 46.5 were male students. Most of the respondents are of 21 to 24 years. The target students are selected from four faculties, namely “Engineering and Technologies,” “Natural Sciences,” “Faculty of Business,” and “Faculty of Law.” The respondents are students enrolled in undergraduate and master’s programs.

## Results

The structural equation modeling (SEM) is applied to test the hypothesis. This section evaluates the reliability and validity of the model proposed by the study. This section also presents the structural model to establish the hypotheses of the study. Measurements and structural models are simulated using the SmartPLS Joe and Hair., 2022.

### Common Method Bias

**FIGURE 3:**
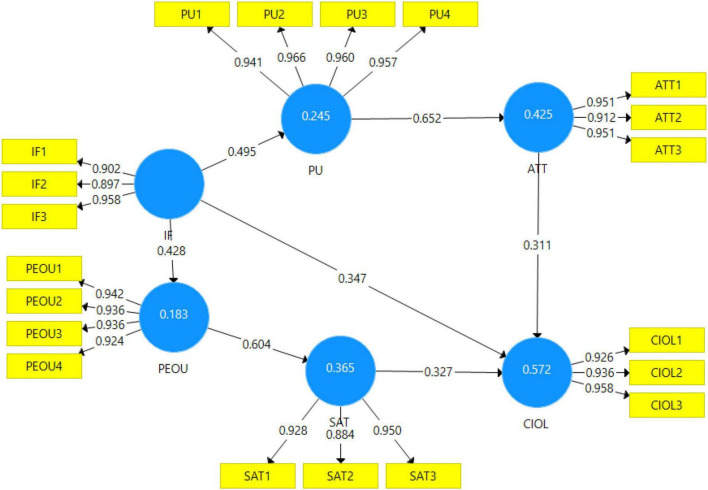
Measurement model.

Inner VIFs values from the collinearity test of the model are also checked for internal reliability. All the inner VIF’s values from the collinearity test are less than 3.3; therefore, the model is free from common method bias. The measurement model is shown in Figure 3, with a factor loading of each item with every construct.

#### Reliability Analysis

The reliability and validity of the model are evaluated using 10% of aggregate data. Cronbach’s alpha test and composite reliability (CR) are used to check the validity through a pilot study. The value of Cronbach’s alpha above 0.7 is high and between 0.7 and 0.35 is acceptable. The six constructs of the study with Cronbach’s alpha values with factor loading are shown in Table 4. For all the constructs, the values of Cronbach’s alpha are higher than 0.7. The values of Cronbach’s alpha for all the constructs are in the acceptable range. The acceptable Cronbach’s alpha values for all the constructs proved the internal consistency of the constructs. In Table 4, the CR of all the constructs is also given which shows the reliability of the study.

**TABLE 4:** Reliability analysis.

Constructs	Items	Factor loading	Cronbach’s Alpha value	CR	AVE
PEU	PEU_1	0.942	0.952	0.965	0.873
	PEU_2	0.936			
	PEU_3	0.936			
	PEU_4	0.924			
PU	PU_1	0.941	0.969	0.977	0.914
	PU_1	0.966			
	PU_1	0.960			
	PU_1	0.957			
SAT	SAT_1	0.928	0.911	0.944	0.848
	SAT_2	0.884			
	SAT_3	0.950			
AT	AT_1	0.951	0.933	0.957	0.881
	AT_2	0.912			
	AT_3	0.951			
IF	IF_1	0.902	0.908	0.942	0.844
	IF_2	0.897			
	IF_3	0.957			
CIOL	CIOL_1	0.936	0.934	0.958	0.884
	CIOL_1	0.926			
	CIOL_3	0.958			

#### Validity Analysis

Validity measures the accuracy of the model to assess the constructs to which extent they measure what they intend to measure. Convergent validity is measured using average value extracted (AVE). The AVE measures the extent to which items converge to measure their corresponding constructs. AVE is the measure of how much variance can be extracted on average from the items to measure constructs, which should be at least 50%. Therefore, a value of AVE above 0.50 is acceptable. From Table 5, it can be observed the value of AVE for all the constructs is above 0.5, and the reliability of the model in terms of AVE is in an acceptable range. Discriminant validity is observed in terms of Fornell-Larcker criteria, cross-loading, and heterotrait–monotrait ratio (HTMT). In Table 6, the square root of the AVE of each construct (highlighted) is higher than all other correlations underneath. Hence, the model validity is proved by Fornell–Larcker criteria. The items loading with their corresponding construct are highlighted. It can be observed that the loading factor of the items with their corresponding construct is higher than with other constructs in the study. The reliability of the model in terms of cross-loading is also proved. The reliability of the model in terms of the heterotrait–monotrait ratio (HTMT) is also given in Table 5, which states that HTMT values of the constructs should be less than 0.85 for a model to be reliable. All the heterotrait–monotrait ratio (HTMT) values for each construct are lower than 0.85, and the model is also reliable in terms of HTMT ratio.

**TABLE 5:** Heterotrait–monotrait ratio.

	AT	CIOL	IF	PEU	PU	SAT
AT						
CIOL	0.539					
IF	0.355	0.675				
PEU	0.489	0.640	0.452			
PU	0.678	0.329	0.523	0.248		
SAT	0.272	0.638	0.590	0.632	0.237	

**TABLE 6:** Fornell–Larcker criterion.

	AT	CIOL	IF	PEU	PU	SAT
AT	**0.938**					
CIOL	0.514	**0.940**				
IF	0.338	0.628	**0.919**			
PEU	0.468	0.607	0.428	**0.934**		
PU	0.652	0.319	0.495	0.240	**0.956**	
SAT	0.264	0.596	0.539	0.604	0.229	**0.921**

*The square root of the AVE of each construct (highlighted) is higher than all other correlations underneath.*

### Structural Model

**FIGURE 4:**
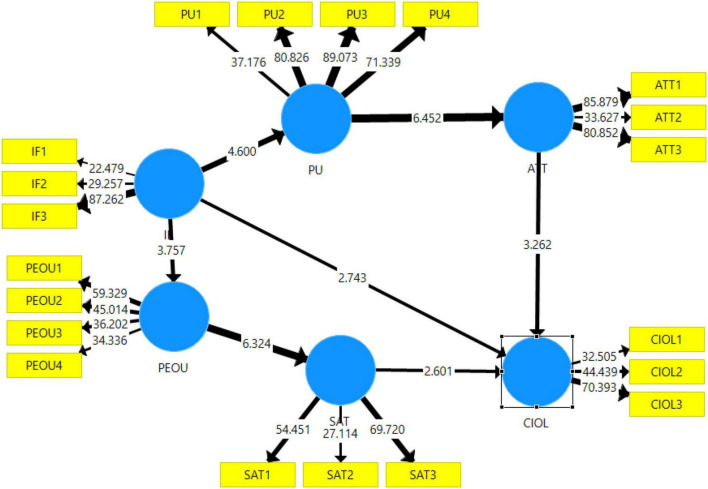
Result of hypothesis testing.

Now, the hypothesis relationship is assessed by the structural model as shown in Figure 4. Each relationship is analyzed in terms of direct, indirect, and total effect of each mediation. The direct relationship is given in Table 7.

**TABLE 7:** Direct relationship.

Path	β	T statistics	*P*-values
IF→PEU	0.428	3.757	0.000
IF→PU	0.495	4.600	0.000
PEU→SAT	0.604	6.324	0.000
SAT→CIOL	0.327	2.601	0.009
PU→AT	0.652	6.452	0.000
AT→CIOL	0.311	3.262	0.001
IF→CIOL	0.347	2.743	0.006

#### Mediation Analysis

To observe the mediation effects of construct on relationships, the mediation relationship is given in Table 8 in the form of indirect relationships. From the statistics given in Table 8, it is observed that IF→PU→AT→CIOL (β = 0.100, *t* = 2.401, *p* = 0.016), IF→PU→AT (β = 0.323, *t* = 4.950, *p* = 0.000), PU→AT→CIOL (β = 0.203, *t* = 3.911, *p* = 0.000), IF→PEU→SAT (β = 0.258, *t* = 2.734, *p* = 0.006), PEU→SAT→CIOL (β = 0.197, *t* = 2.325, *p* = 0.020) play a significant mediation role while the path from immediacy of feedback to Perceived Ease of Use to satisfaction has no significant mediation role on relationship between immediacy of feedback to continuous intentions to use online learning. However, the total effects derived by derived and indirect effects are given in Table 9, which reveals that the Hypothesis 1, IFF→PEU (β = 0.428, *t* = 3.757, *p* = 0.000), Hypothesis 2, IF→PU (β = 0.495, *t* = 4.600, *p* = 0.000), Hypothesis 3, PEU→SAT (β = 0.604, *t* = 6.324, p = 0.000), Hypothesis 4, SAT→CIOL (β = 0.327, *t* = 2.601, p = 0.009), Hypothesis 5, PU→AT (β = 0.652, *t* = 6.452, p = 0.000), Hypothesis 6, AT→CIOL (β = 0.311, *t* = 3.262, p = 0.001), and Hypothesis 7, IFF→CIOL (β = 0.347, *t* = 2.743, *p* = 0.006) are also accepted.

**TABLE 8:** Indirect relationship.

Path	β	T statistics	*P*-values
IF→PU→AT→CIOL	0.100	2.401	0.016
IF→PU→AT	0.323	4.950	0.000
PU→AT→CIOL	0.203	3.911	0.000
IF→PEU→SAT→CIOL	0.084	2.987	0.003
IF→PEU→SAT	0.258	2.734	0.006
PEU→SAT→COIL	0.197	2.325	0.020

**TABLE 9:** Total effect.

Path	β	T statistics	*P*-values
IF→PEU	0.428	3.757	0.000
IF→PU	0.495	4.600	0.000
PEU→SAT	0.604	6.324	0.000
SAT→CIOL	0.327	2.601	0.009
PU→AT	0.652	6.452	0.000
AT→CIOL	0.311	3.262	0.001
IF→CIOL	0.532	5.107	0.000

#### Impact of Moderator

This study also explores the impact of problem-solving skills on students’ continuous intentions to use online learning. To explore, the problem-solving skills of the students are used as a moderator. The effect of the moderator on the continuous intentions to use online learning with media immediacy of feedback is explored. The results are shown in Table 10. The improvements in the insignificance of the existing relationship proved to accept the Hypothesis that Student problem-solving skills will significantly enhance the positive relationship between student’s attitude toward online systems and students’ perceived usefulness of the online learning system.

**TABLE 10:** Impact of moderator.

	Path	β	T statistics	*P*-values
H8	IF→CIOL	0.448	3.857	0.000

## Discussion and Conclusion

This study explores the impact of the immediacy of feedback on students’ continuous intentions to use online learning. This study uses the technology acceptance model (TAM) and information system continuance (IS Continuance) theory. IS continuance is the extension of ECT theory to determine the impact of user satisfaction, confirmation, and Perceived Ease of Use (PEU) on user continuous intentions to use an information system. Both the IS and TAM theories are also used as mediation effects on the immediacy of feedback from media and student continuance intention to use online learning, which is explored in the next section.

Initially, the IS continuance theory is used to determine the impact of the immediacy of feedback (IF) on the continuous intentions to use online learning (CIOL). The impact of the immediacy of feedback on the Perceived Ease of Use (PEU) of online learning is determined by Hypothesis 1. Hypothesis 1 states that (IF→PEU) *“Immediacy of feedback in online learning is positively correlated with the student Perceived Ease of use (PEU) of online learning.*” The statistics (β = 0.428, *t* = 3.757, *p* = 0.000) after data analysis reveals accept hypothesis 1 that immediacy of feedback is positively correlated with the student’s perceived Ease of Use (PEU) of the online learning system. Once Hypothesis 1 is established, the next is to explore the impact of PEU on online learning on students’ satisfaction with online learning. The impact of PEU on online learning is explored by Hypothesis 2 which states that “*student Perceived Ease of Use (PEU) of online learning system is positively correlated with the student’s satisfaction with online learning.*” The statistics related to hypothesis 3, PEU→SAT, (β = 0.604, *t* = 6.324, *p* = 0.000) reveal to accept hypothesis 2. The PEU relationship with the student’s satisfaction with online learning is established. The next is to explore the relationship between the student’s satisfaction with online learning and the student’s continuous intentions to use the online learning system. For this purpose, Hypothesis 4 (H4) states that “*student’s satisfaction of online learning is positively correlated with students’ continuous intentions to use online learning system*” is tested. The statistics related to Hypothesis 4, SAT→CIOL (β = 0.456, *t* = 3.567, *p* = 0.001), reveal to accept the hypothesis 4. The indirect effect of mediation IF→PEU→SAT→CIOL (β = 0.084, *t* = 2.987, *p* = 0.003) reveals that the mediation path of Immediacy of feedback to Perceived Ease of Use (PEU) to satisfaction and continuous intentions to use online learning is also significant. The findings are in line with the previous study (Lee, 2010; Abdul Rahman et al., 2020; Al-Maroof et al., 2020; Wan et al., 2020; Chen et al., 2021)

Now, the TAM theory is used to determine the impact of immediacy of feedback on students’ continuous intentions to use online learning through Perceived Usefulness (PU) and attitude. The impact of the immediacy of feedback is explored through students’ Perceived Usefulness (PU) of online learning and students’ attitude toward the continuous intentions to use online learning. Initially, the relationship between the immediacy of feedback with the student’s perceived Usefulness (PU) of online learning is explored through hypothesis 2. Hypothesis 2 (H2) states that “*Immediacy of feedback in online learning is positively correlated with the student Perceived usefulness (PU) of the online learning.*” Hypothesis 2 is accepted with statistics IF→PU (β = 0.495, *t* = 4.600, *p* = 0.000). Once hypothesis 2 is established, the next is to explore the impact of the student’s Perceived Usefulness (PU) of online learning on the student’s attitude toward the online learning system. The relationship between the student’s PU toward attitude is determined by hypothesis 5. Hypothesis 5 (H5) states that “*student Perceived Usefulness (PU) of online learning online learning system is positively correlated with the student’s Attitude toward online learning.*” The statistics related to hypothesis 5 (H5), PU→AT (β = 0.652, *t* = 6.452, *p* = 0.000), reveal to accept the hypothesis 5. With the establishment of hypothesis 5, the next step is to explore the relationship between students’ attitudes toward online learning and students’ continuous intentions to use online learning. The relationship is explored by hypothesis. Hypothesis 6 (H6) states that “*student’s Attitude toward online system has a positive relationship with student’s continuous intentions to use online learning.*” The statistics related to hypothesis 6, AT→CIOL (β = 0.311, *t* = 3.262, *p* = 0.001), reveal to accept the hypothesis. Hypothesis 6 is established and reveals that there is a significant positive relationship between the student attitude toward online learning and students’ continuous intentions to use online learning. The mediation effect of students’ Perceived Usefulness (PU) and students’ attitude toward continuous intentions to use online learning is significant. The mediation effect of IF→PU→AT→CIOL is significant and plays a positive role in continuous intention to use online learning with the immediacy of feedback.

The next is to explore the direct relationship between the immediacy of feedback on the student’s continuous intentions to use online learning. The relationship between the immediacy of feedback and continuous intentions to use online learning is described by Hypothesis 7. Hypothesis 7 (H7) states that “*Immediacy of feedback of media has a significant relationship with students’ continuous intentions to use online learning.*” The statistics related to H7, IF→CIOL (β = 0.347, *t* = 2.743, *p* = 0.006), reveal to accept the hypothesis. This is stated in terms of hypothesis 8. Hypothesis 8 (H8) states that “*student problem-solving skills will significantly enhance the positive relationship immediacy of feedback of media and student’s continuous intentions to use online learning.*” The statistics related to hypothesis 8, IFF→CIOL (β = 0.347, *t* = 2.743, *p* = 0.006), reveal that there is a significant relationship between the student’s problem-solving skills on the student’s continuous intentions to use online learning with the immediacy of feedback in online learning. The results explored in this study also agree with existing studies. The findings related to continuous intentions to use online learning are in line with findings of Wang et al. (2021).

The study explores the relationship between the immediacy of feedback from media on continuous intentions to use online learning in future from students’ perspectives. The relationship is explored directly as well as through different mediation roles of immediacy feedback on Perceived Ease of Use (PEU), Perceived Usefulness (PU), satisfaction, attitude, and finally the continuous intentions to use online learning. The eight hypotheses were made using a conceptual model based on technology acceptance model (TAM) and information system continuance theory. The survey was conducted with the target graduate and master’s level students. The measurement model is reliable in both convergent and discriminatory validity. The structural model reveals that all the hypotheses from H1 to H7 are accepted based on *T*-test and *P*-values. It is observed that students’ problem-solving skills have a significant relationship between the immediacy of feedback and continuous intentions to use online learning. The effect of immediacy on continuous intentions to use online learning is significant, and the relationship is improved with student problem-solving skills. The findings of the study that immediacy of feedback is positively related with PEOU and PU that are positively related with attitude and satisfaction that are positively associated with continuous intentions to use online learning are in accordance with findings of the previous studies (Almahamid and Rub, n.d.; Daneji et al., 2019; Ashrafi et al., 2020; Dai et al., 2020; Gupta et al., 2020).

## Implication, Limitation, and Future Research

### Theoretical Implications

This study offers two contributions that help to advance the evolution of online learning. First, the positive impacts of the immediacy of feedback from media on student continuous intentions to use online learning. Second, this study adds to the scant literature on the impacts of the immediacy of feedback characteristic of media on student continuous intentions to use online learning from the student perspective, by empirical investigation. Studies were conducted to explore the students’ perceptions about online learning (Mehta and Shah, 2022) as compared to face-to-face learning and different factors affecting the success of online learning, but the impact of the immediacy of feedback is not explored yet. The previous literature focuses on the identification of factors that affects the students’ continuous intentions to use online learning (Al-Rahmi et al., 2019). Many studies are conducted to explore the success factors of online learning, but the impact of the immediacy of feedback is not explored by any study.

The second contribution is the impact of the immediacy of feedback on continuous intentions to use online learning through different mediation roles of PEOU, PU, satisfaction, and attitude toward continuous intentions to use online learning. Many studies apply the TAM model to explore success factors for the adaptation of E-learning with different moderating roles (Liao et al., 2022), but the conceptual model is unique in terms of exploration of the unique attribute of media that has not been previously targeted. The third contribution is to explore the impacts of student problem-solving skills over the previously discovered association as the moderator. The simultaneous impact of media attributes and student personal skills on continuous use of online learning is not explored before. The findings of the study can be used as the basis of future research by exploration and extension of the proposed model. Advancements in our research provide insight for the researcher to explore the new attributes of the media that would be important for the success of online learning in future to take inherent advantages associated with online learning.

Moreover, this study explores the relationship between the immediacy of feedback from media on students’ continuous intentions to use online learning in terms of existing models and theories. The result of this study also strengthens the existing model and paves the way toward the development of a new model to explore the impacts of media richness on students’ continuance intention to use online learning. Apart from media richness, this study also stresses the need for other factors that strengthen the intention to continuously use online learning that would determine the success of online learning in future.

### Practical Implications

This study has many practical implementations for the designers, developers, and teachers. This study stresses the importance of immediacy of the feedback on the success of online learning in terms of students’ intentions to use online learning. In the past, different efforts were made to explore the directions of improvements in online learning (Fry, 2001).

The impacts of human–computer interaction (HCI) on the success of online learning are explored (Alebeisat et al., 2022). This model provides insight for the designer of online learning to emphasize the role of the immediacy of feedback in the online learning system for their acceptance from the students in future. The model would be helpful for the technologists to adopt the interaction of the students in terms of immediacy of feedback from the student. The model would also provide insight for the teachers to design courses with due immediacy of feedback for successful adoption of online learning by the students. Overall, this study provides a model to realize the importance of immediacy of feedback in the success of online learning by students’ continuous intentions to use online learning in future.

This study also identifies the important factor in the success of online learning. The findings of the study provide insight for the technologists and course designers to take care of the importance of the immediacy of feedback. The identified findings are important in that it determines the success of online learning. To motivates students for their continuous intentions to use online learning, the immediacy of feedback is important for the successful implementation of the online learning program.

### Limitations and Further Research

This study explores the impact of the immediacy of feedback on continuous intentions to continuously use online learning from the student’s perspective. The continuous intention to use online learning determines the success of online learning. The study explores the impacts from a student perspective only. The teacher’s intentions to use online learning are also important that are not explored due to the limited scope of the study. There is a need to explore the intentions of the teachers to use online learning in future with the immediacy of feedback, which is not part of the study. There is also a need to assess the impacts of the immediacy of feedback on continuous intentions to use online learning with other important variables like content, students’ motivations, and students’ involvement.

## Data Availability Statement

The original contributions presented in the study are included in the article/Supplementary Material, further inquiries can be directed to the corresponding author/s.

## Ethics Statement

The studies involving human participants were reviewed and approved by the Research Ethics Committee of College of Art and Design, Shangqiu Normal University, Shangqiu. The patients/participants provided their written informed consent to participate in this study.

## Author Contributions

Both authors contributed substantially in the published version of the manuscript.

## Conflict of Interest

The authors declare that the research was conducted in the absence of any commercial or financial relationships that could be construed as a potential conflict of interest.

## Publisher’s Note

All claims expressed in this article are solely those of the authors and do not necessarily represent those of their affiliated organizations, or those of the publisher, the editors and the reviewers. Any product that may be evaluated in this article, or claim that may be made by its manufacturer, is not guaranteed or endorsed by the publisher.
