# Supplementary material for: Impact of Immediacy of Feedback on Continuous Intentions to Use Online Learning From the Student Perspective
Source: Front Psychol. 2022 Jun 30;13:865680. doi: 10.3389/fpsyg.2022.865680 (PMC9280469; doi:10.3389/fpsyg.2022.865680)
Supplement: Supplementary file 2 [file Table_2.DOCX]

Appendix A


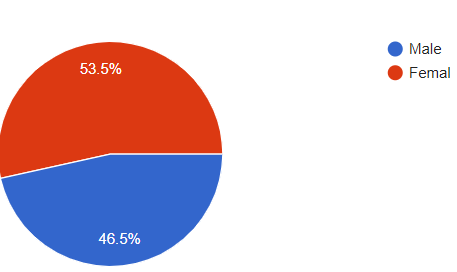


Figure 1. Male-female respondent ratio


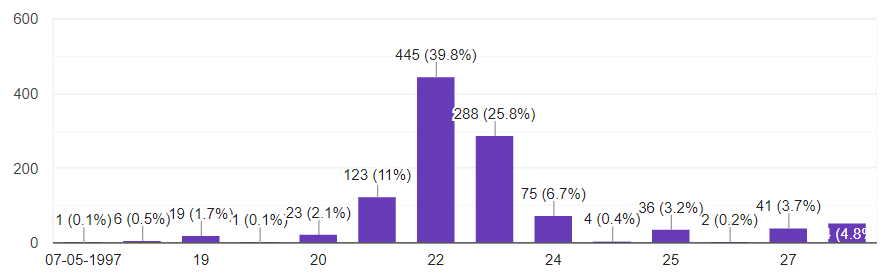


Figure 2. Age and responses

**Table 1.** Constructs with items

| Constructs | Number of Items | Source |
| --- | --- | --- |
| Perceived Ease of Use (PEU) | 4 | [19] |
| Satisfaction (SAT) | 3 | [30]–[32] |
| Attitude (AT) | 3 | [30]–[32] |
| The immediacy of Feedback (IF) | 3 | [32], [49], [50] |
| Continuous Intentions to use online Learning (CIOL) | 3 | [32], [52] |
| Perceived Usefulness (PU) | 4 | [19], [32] |

**Table 2.** Questionnaire items

| Constructs | Items |
| --- | --- |
| Perceived Ease of Use (PEU) | The online learning system is easy |
|  | It is easy to do my education activities with the online learning system |
|  | I have a complete understanding of the online learning system |
|  | In general, online learning is easy to use. |
| Perceived Usefulness (PU) | Online learning progresses performance in learning |
|  | Online Learning improved learning productivity |
|  | Online learning is useful than traditional learning |
|  | Overall, online learning is useful in learning |
| Satisfaction (SAT) | Online learning provides a complete learning environment |
|  | In general, I am quite satisfied with online learning |
| Attitude (AT) | I like online learning |
|  | Online learning is fun to use |
|  | Online learning is an attractive learning environment |
| Continuous Intentions to use online Learning (CIOL) | I intend to use online learning in future |
|  | In the future, I desire to use online learning rather than the traditional model of learning |
|  | I have a choice I will go online for all my courses |

Appendix B

**Table 3.** Inner VIF’s values from collinearity test of the model

|  | PEU | PU | SAT | AT | IF | CIOL |
| --- | --- | --- | --- | --- | --- | --- |
| PEU |  |  | 1.000 |  |  |  |
| PU |  |  |  | 1.000 |  |  |
| SAT |  |  |  |  |  | 1.425 |
| AT |  |  |  |  |  | 1.141 |
| IF | 1.000 | 1.000 |  |  |  | 1.498 |

**Table 4.** Cross Loading

|  | AT | CIOL | IF | PEU | PU | SAT |
| --- | --- | --- | --- | --- | --- | --- |
| AT_1 | 0.951 | 0.551 | 0.372 | 0.469 | 0.637 | 0.306 |
| AT_2 | 0.912 | 0.381 | 0.224 | 0.334 | 0.521 | 0.172 |
| AT_3 | 0.951 | 0.494 | 0.337 | 0.494 | 0.662 | 0.248 |
| CIOL_1 | 0.529 | 0.926 | 0.594 | 0.628 | 0.352 | 0.600 |
| CIOL_2 | 0.419 | 0.936 | 0.547 | 0.503 | 0.241 | 0.509 |
| CIOL_3 | 0.493 | 0.958 | 0.625 | 0.570 | 0.297 | 0.564 |
| IF_1 | 0.364 | 0.589 | 0.902 | 0.477 | 0.479 | 0.429 |
| IF_2 | 0.240 | 0.509 | 0.897 | 0.290 | 0.407 | 0.497 |
| IF_3 | 0.315 | 0.622 | 0.958 | 0.393 | 0.472 | 0.565 |
| PEU_1 | 0.453 | 0.593 | 0.416 | 0.942 | 0.270 | 0.600 |
| PEU_2 | 0.427 | 0.559 | 0.361 | 0.936 | 0.208 | 0.519 |
| PEU_3 | 0.440 | 0.545 | 0.396 | 0.936 | 0.206 | 0.627 |
| PEU_4 | 0.429 | 0.571 | 0.424 | 0.924 | 0.211 | 0.499 |
| PU_1 | 0.607 | 0.321 | 0.483 | 0.230 | 0.941 | 0.198 |
| PU_2 | 0.599 | 0.250 | 0.436 | 0.213 | 0.966 | 0.199 |
| PU_3 | 0.667 | 0.364 | 0.528 | 0.267 | 0.960 | 0.268 |
| PU_4 | 0.615 | 0.274 | 0.439 | 0.204 | 0.957 | 0.203 |
| SAT_1 | 0.300 | 0.555 | 0.538 | 0.636 | 0.236 | 0.928 |
| SAT_2 | 0.141 | 0.477 | 0.427 | 0.440 | 0.146 | 0.884 |
| SAT_3 | 0.266 | 0.602 | 0.514 | 0.571 | 0.237 | 0.950 |

**Table 5.** Hetrotrait-Monotrait ratio (HTMT)

|  | AT | CIOL | IF | PEU | PU | SAT |
| --- | --- | --- | --- | --- | --- | --- |
| AT |  |  |  |  |  |  |
| CIOL | 0.539 |  |  |  |  |  |
| IF | 0.355 | 0.675 |  |  |  |  |
| PEU | 0.489 | 0.640 | 0.452 |  |  |  |
| PU | 0.678 | 0.329 | 0.523 | 0.248 |  |  |
| SAT | 0.272 | 0.638 | 0.590 | 0.632 | 0.237 |  |

Appendix C

**Table 6.** Total Effect

| Path | β | T statistics | P values |
| --- | --- | --- | --- |
| IF🡪PEU | 0.428 | 3.757 | 0.000 |
| IF🡪PU | 0.495 | 4.600 | 0.000 |
| PEU🡪SAT | 0.604 | 6.324 | 0.000 |
| SAT🡪CIOL | 0.327 | 2.601 | 0.009 |
| PU🡪AT | 0.652 | 6.452 | 0.000 |
| AT🡪CIOL | 0.311 | 3.262 | 0.001 |
| IF🡪CIOL | 0.532 | 5.107 | 0.000 |
